# Identifying the role of the reticulospinal tract for strength and motor recovery: A scoping review of nonhuman and human studies

**DOI:** 10.14814/phy2.15765

**Published:** 2023-07-20

**Authors:** Yonas Akalu, Ashlyn K. Frazer, Glyn Howatson, Alan J. Pearce, Ummatul Siddique, Mohamad Rostami, Jamie Tallent, Dawson J. Kidgell

**Affiliations:** ^1^ Monash Exercise Neuroplasticity Research Unit Department of Physiotherapy School of Primary and Allied Health Care, Faculty of Medicine, Nursing and Health Science Monash University Melbourne Victoria Australia; ^2^ Department of Human Physiology School of Medicine University of Gondar Gondar Ethiopia; ^3^ Department of Sport, Exercise and Rehabilitation Northumbria University Newcastle UK; ^4^ Water Research Group North West University Potchefstroom South Africa; ^5^ College of Science, Health and Engineering La Trobe University Melbourne Victoria Australia; ^6^ School of Sport, Rehabilitation and Exercise Sciences University of Essex Colchester UK

**Keywords:** maximum force production, motor recovery, reticulospinal tract, scoping review, strength

## Abstract

In addition to the established postural control role of the reticulospinal tract (RST), there has been an increasing interest on its involvement in strength, motor recovery, and other gross motor functions. However, there are no reviews that have systematically assessed the overall motor function of the RST. Therefore, we aimed to determine the role of the RST underpinning motor function and recovery. We performed a literature search using Ovid Medline, Embase, CINAHL Plus, and Scopus to retrieve papers using key words for RST, strength, and motor recovery. Human and animal studies which assessed the role of RST were included. Studies were screened and 32 eligible studies were included for the final analysis. Of these, 21 of them were human studies while the remaining were on monkeys and rats. Seven experimental animal studies and four human studies provided evidence for the involvement of the RST in motor recovery, while two experimental animal studies and eight human studies provided evidence for strength gain. The RST influenced gross motor function in two experimental animal studies and five human studies. Overall, the RST has an important role for motor recovery, gross motor function and at least in part, underpins strength gain. The role of RST for strength gain in healthy people and its involvement in spasticity in a clinical population has been limitedly described. Further studies are required to ascertain the role of the RST's role in enhancing strength and its contribution to the development of spasticity.

## Graphical abstract

Schematic overview of the physiological role of the reticulospinal tract in motor function

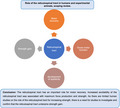

## 1 INTRODUCTION

Strength is defined as the maximal amount of torque that can be produced by a muscle (Coulson, 2021). It is improved by physical activity and is associated with a 10%–17% decrease in all‐cause mortality, total cancer, diabetes, and cardiovascular diseases (Momma et al., 2022). Being active is an integral part of life which improves quality of life and increases life expectancy (National Institute on Aging, 2001; Schoenfeld et al., 2021). Globally, 9% of premature deaths and 6%–10% of major chronic noncommunicable diseases are caused by physical inactivity (Lee et al., 2012). Strength training reduces the risk of depression (Salmon, 2001); minimizes muscle loss during aging (Tieland et al., 2018); is effective for reducing chronic pain (Geneen et al., 2017); and is used for rehabilitation or recovery from disability following stroke and orthopedic surgery (Ada et al., 2006) among others. Strength gain leads to functional independence, improved cognition and self‐esteem (Westcott, 2012). Moreover, strength training is important for increasing performance in different populations (Breese, 2019; Haff & Triplett, 2015). A decrease in strength leads to muscle weakness and consequent functional impairment (Visser et al., 2000) or disability (Rantanen et al., 1999), increased dependency (Janssen et al., 2002), reduced quality of life (Clark et al., 2015), and higher risk of fall injury (De Rekeneire et al., 2003) and mortality (Newman et al., 2006). Therefore, it is imperative to prevent the loss of strength, and strength training has been shown to be an effective intervention to counteract the decline in the force generating capacity of the muscle because of injury, disease, or aging (Siddique et al., 2022).

Strength gain, a state of getting stronger overtime (Glover & Baker, 2020), occurs due to both muscular and neural adaptations (Folland & Williams, 2007). Strength begins to increase following three to five training sessions (Del Vecchio et al., 2019; Hortobágyi et al., 2011; Mason et al., 2020), and these immediate strength gains within the first few weeks are attributed to neural adaptations (Pearcey et al., 2021). Neural adaptations that underpin strength gain likely involve multiple sites in the nervous system (Gabriel et al., 2006; Lee et al., 2009). However, the precise site of adaptation remains relatively ambiguous, although changes in intracortical inhibition seem to be commonly reported in many studies (Škarabot et al., 2021). Increased corticospinal excitability (CSE) following strength training has been reported by some studies (Goodwill et al., 2012; Mason et al., 2017) while other studies have reported no changes (Ansdell et al., 2020; Jensen et al., 2005; Latella et al., 2012). A meta‐analysis by Kidgell et al. (2017) and a study on humans by Nuzzo et al. (2017) showed that adaptation following strength training involves reduced short‐interval cortical inhibition (SICI) and cortical silent period but no changes in CSE. Many of the studies have focused on the motor cortex and corticospinal tract (CST), and the findings are inconsistent regarding the predominant site of neural adaptation (Atkinson et al., 2022). Interestingly, studies that have used a metronome or controlled the repetition timing (i.e., externally paced strength training) have reported increases in CSE and reductions in SICI, while studies that have included self‐paced strength training have reported no changes in CSE or SICI (Leung et al., 2015). More importantly, whether the strength training is paced or not, strength gain still occurs (Leung et al., 2015). Therefore, there must be other sites, for instance the reticulospinal tract (RST), which might account for the increase in strength in the absence of changes in CSE and SICI (Škarabot et al., 2021). In support of this, nonhuman studies have reported that the RST is a potential site of neural adaptation to strength training (Glover & Baker, 2020).

The RST, the most important extrapyramidal tract, is a major descending pathway primarily responsible for locomotion and postural control (Prentice & Drew, 2001; Schepens & Drew, 2006). Additionally, the RST regulates muscle tone during gait (Takakusaki et al., 2016) and controls upper limb muscle activity (Dean & Baker, 2017). In contrast to the dominant contralateral CST that innervate smaller motoneuron pools (Buys et al., 1986) responsible for fine movements (Zaaimi et al., 2018), the RST operates bilaterally in the spinal cord, supplying larger groups of neurons in a synergistic pattern (Peterson et al., 1975). The involvement of the RST in gross motor function has been confirmed through studies conducted on nonhuman subjects, such as macaque monkeys. These studies have shown that surgical lesions to the CST lead to the loss of fine movement, whereas lesions to the RST result in the loss of gross motor function. Importantly, recovery of gross motor function has been observed following a lesion to the RST (Lawrence & Kuypers, 1968).

The RST also has a role for the recovery of gross motor functions following contralateral CST lesioning. Zaaimi et al. (2012) showed that post‐recovery medial longitudinal fasciculus (MLF) stimulation, a technique to assess the RST function, resulted in increased amplitude of postsynaptic potentials elicited from motoneurons innervating forearm flexor muscles while ipsilateral pyramidal stimulation resulted in weak responses in forearm and hand muscles (Zaaimi et al., 2012), implying the important role of the RST in functional motor recovery.

In addition to the studies on nonhumans, stroke patients also showed the role of RST in gross motor function and resistance training (Alagona et al., 2001; Li et al., 2019; Pineiro et al., 2001). In stroke patients with a lesion to the CST, the RST undergoes adaptation involving increased synaptic efficacy, and strengthens its connectivity to preserve motor function or compensate for the loss of motor function of the CST (Alagona et al., 2001; Li et al., 2019; Pineiro et al., 2001). In patients with spinal cord injury (SCI), there was increased excitability of the reticular system to partly restore motor function in the upper limb, compensating for injury to the spinal cord (Baker & Perez, 2017; Sangari & Perez, 2019). Taken together, in experimental animals and in humans, the excitability of the RST appears to be important for the recovery of motor function following injury and/or disease.

Despite the absence of reviews that have systematically assessed the overall motor function of RST, the limited number of studies available and their heterogeneity pose challenges in synthesizing the data for a meta‐analysis. Therefore, we conducted a scoping review with the aim to systematically assess the overall motor function of the RST. Specifically, this scoping review, appraised all experimental animal and human studies on the role of the RST in strength gain, gross motor function, development of spasticity, and motor recovery following neurological injury. The findings of this review will provide a synthesis of information on the RST and guide future efforts to investigate the RST in the context of strength training and motor recovery.

## 2 MATERIALS AND METHODS

### 2.1 Search strategy

This scoping review followed the Preferred Reporting Items for Systematic Reviews and Meta‐Analyses extension for Scoping Reviews (the PRISMA‐ScR) reporting guideline. The search was performed using four databases: Ovid Medline, Embase, CINAHL Plus, and Scopus to retrieve papers for this scoping review. For the search, key terms were used for our main objective or research question to access important studies. Alternative terms for each key word were also searched in the index terms, title, and abstract of each database. Search results of each key word and their alternative terms were combined by the Boolean operator “OR”, and the sets of key words and their alternative terms were combined by the Boolean operator “AND”. We limited our search to English language. The search strategy for OVID Medline and Embase databases (The search strategies for all used databases are attached as Data S1) were ((“Reticulospinal tract*” OR “Reticulospinal outflow*” OR“Ipsilateral motor‐evoked potential*” OR “Ipsilateral MEP*” OR “Acoustic startle*” OR “StartReact”*) and ((“Hand strength*” OR “Muscle strength*” OR “Muscle force*” OR “Maximum voluntary contraction*” OR “Balance*” OR “Motor Recover*” OR “voluntary elbow flexion*” OR “Grip strength*” OR “Sarcopenia” OR “Isometric contraction*” OR “wrist flexor muscle*” OR “hand muscle*” OR “dorsal interosseous muscle*”).

### 2.2 Study eligibility criteria

#### 2.2.1 Types of participants

All studies which focused on humans aged older than 18 and on experimental animals involved in the assessment of the role of RST were included.

#### 2.2.2 Concept

Studies on the function of and changes in the connectivity of the RST during different circumstances including motor recovery (i.e., restoration of motor performance following insult to the nervous system), strength training and aging were included.

#### 2.2.3 Context

Studies conducted on experimental animals in the laboratory or in human subjects, male or female, in the community undergoing strength training or without strength training, on patients with SCI or stroke resulting in muscle weakness or motor impairment were included in this scoping review. Studies that did not assess or report the RST changes or function were excluded.

### 2.3 Types of evidence sources

Primary studies, quantitative or qualitative, were included in this scoping review. Systematic and narrative reviews were excluded but their reference lists were checked manually for any relevant study.

### 2.4 Evidence screening and selection

Selected studies for the scoping review were based on our inclusion and exclusion criteria. Any study on nonhuman or human reporting on the role of RST was included. No publication year or geographical restriction was applied. Studies written in English and published before our search, 20 March 2023, were included. All search results were transferred to Endnote Version 20 reference manager and then exported to Covidence to remove duplicates and for title and abstract screening, and full‐text review by two independent reviewers (YA and DJK). During full‐text review, studies with an appropriate design (to answer our research questions) and reporting or assessing the change, role, contribution, and effect of the RST were considered relevant and selected for the final data extraction. Studies which did not clearly and specifically state the role of RST were excluded. Five studies were excluded during full‐text review due to: wrong outcome/different outcome of interest (Fujiyama et al., 2011); failure to state the role of RST for strength or motor function/motor recovery (Chen et al., 2016), inaccessible paper (Choudhury et al., 2017); lack of clarity whether the sound elicited a StartReact response and the intensity of sound not known (Aluru et al., 2014); nonspecific report on RST, rather on extrapyramidal tract contribution for motor impairment (Paul et al., 2022). During the screening process, disagreements were resolved by a third reviewer (MR).

### 2.5 Data charting

After selection of relevant studies, the following data were extracted using Microsoft Excel: author name, country, publication year, population type (experimental animal [Rat or monkeys] or human, healthy participant or patients with stroke, SCI), sample size, aim of the study, intervention, motor tasks, type of muscle used for electromyography (EMG) recording, target muscle or nerve for intervention for interventional studies, measurement method of outcome variable or technique of RST assessment (e.g., StartReact, ipsilateral motor‐evoked potential [iMEP] or other) and the role of RST or key electrophysiological findings.

## 3 RESULTS

### 3.1 Search results and studies' characteristics

**FIGURE 1:**
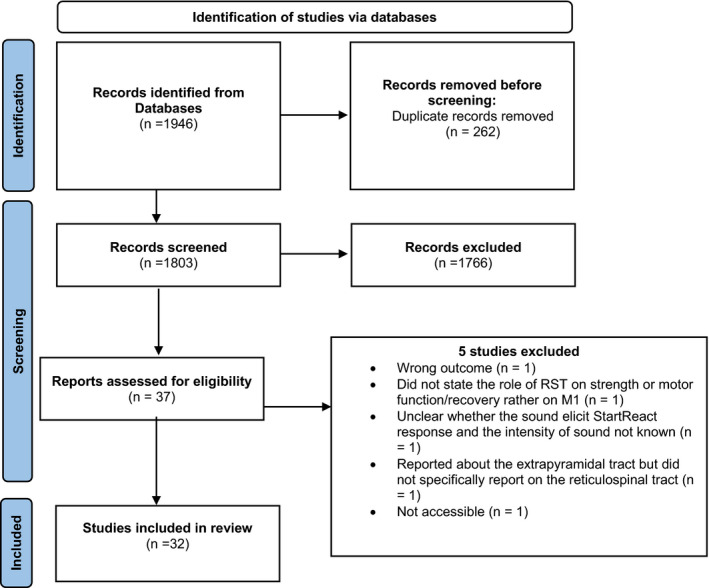
The process of identifying, screening, and assessing the included studies according to the PRISMA‐ScR 2018 guidelines. M1: Primary motor cortex; RST: Reticulospinal tract.

A total of 1946 studies were accessed using Medline (1618), Embase (156), CINAHL (Valls‐Sole, 2012), and Scopus (172) databases. We also acquired 30 additional papers directly from reference lists of review papers. After removal of duplication (262 papers), 36 relevant studies were identified by title and abstract screening. From these relevant studies, four were excluded during full‐text review. One of the relevant studies was inaccessible and hence excluded (Choudhury et al., 2017). Finally, 32 papers were included for the analysis (Figure 1 PRISMA). From the total of 32 studies, 22 were human studies (Tables 1, 2, 3, 4).

**TABLE 1:** Study characteristics for included studies within the scoping review.

S.no	Author, year of publication	Country	Type of population	Sample Size	Aim of the study	Type of muscles for EMG measurement, or target central nervous system structure involved in the study
1	Alagona et al. (2001)	Italy	Stroke patients and Healthy controls	41 (Patients: 25 Controls: 16)	To compare the prevalence and characteristics of ipsilateral upper limb MEPs in a population of healthy subjects and acute‐stroke patients and to determine whether the presence of ipsilateral responses in acute‐stroke patients contributes to recovery.	FDI or biceps brachii, finger extensor and triceps brachii muscles.
2	Anzak, Tan, Pogosyan, & Brown, (2011)	UK	Healthy participants	18	To determine whether a loud auditory stimulus improves response force over and above that possible through the maximum effort of will.	SCM ipsilateral to the tested hand.
3	Anzak, Tan, Pogosyan, Djamshidian, et al. (2011)	UK	Parkinson's disease patient and healthy controls	18 (patients: 9, controls: 9)	Effect of dopaminergic state (on and off treatment state) on any improvements in the magnitude and rate of force development following intense auditory stimuli.	SCM
4	Baker & Perez, (2017)	UK	Spinal cord injury (SCI) patients and healthy controls	39 (SCI: 17; controls: 22)	To examine the preferential contribution of the RST following SCI to gross hand function or independent finger movements.	Intrinsic finger muscle, SCM.
5	Ballermann & Fouad, (2006)	Canada	Rat	25 (lesioned: 21; controls: 4)	To confirm sprouting of the RST with collaterals crossing the midline promotes locomotion and delayed recovery.	Gigantocellular division of the medullary reticular formation and spinal cord.
6	Choudhury et al. (2019)	India	Stroke patients and heathy controls	114 (stroke: 95; controls: 19)	Evaluating the marker of reticulospinal output in stroke survivors with varying degrees of motor recovery.	Wrist flexor Muscles.
7	Colomer‐Poveda et al. (2023)	Spain	Resistance‐trained rock climbers and untrained individuals	31 (16 elite level rock climbers and 15 non‐trained)	To determine if chronic rock climbing and climbing‐specific resistance training would modify the efficacy of the RST.	FDS and EDC of right limb.
8	Coppens et al. (2018)	Netherland	Stroke patients and healthy controls	24 (stroke: 12; controls: 12)	To investigate the StartReact effect on APRs.	TA, rectus femoris, and SCM.
9	Darling et al. (2018)	USA	Rhesus monkeys (*Macaca mulatta*)	13 (lesioned: 9 controls: 5)	Investigate the cortico‐reticular mechanism underlying variable recovery of fine hand/digit motor function in monkeys with frontoparietal lesions.	Brain stem (Gigantocellular reticular nucleus of the medulla), M1, lateral premotor cortex, primary somatosensory cortex, anterior parietal cortex.
10	Engmann et al. (2020)	Switzerland	Female Lewis rats	20 (lesioned: 13 controls: 7)	Investigate the functional relevance of locally‐rewired versus compensatory Gigantocellular reticularis plasticity for the recovery of overground locomotion observed after spinal hemisection.	Brain stem (Gigantocellular reticular area) and spinal cord.
11	Fernandez‐Del‐Olmo et al. (2014)	Spain	Trained human subjects	13	To investigate the effects of auditory stimuli of different intensities on the rate of force development and reaction time, during the performance of a concentric bench‐press exercise in subjects with resistance training experience, using kinematic and electromyography parameters.	Pectoralis major, triceps brachii and SCM.
12	Glover & Baker, (2020)	UK	Female macaque monkey	2	Compare the relative contributions of intracortical, corticospinal, and reticulospinal networks to the neural adaptations associated with strength training.	FDI, FDS, FCR, EDC, biceps brachii, triceps brachii, PMJ, and PDLT.
13	Glover & Baker, (2022)	UK	Female rhesus macaque monkeys	2	To compare the coding of force for the reticular formation and corticospinal cells in macaque monkeys trained to perform a weight‐lifting task.	Eight upper limb muscles: FDI, FDS, FCR, EDC, biceps brachii; triceps brachii, PMJ, and PDLT.
14	Grosse & Brown, (2003)	Germany	Healthy subjects	15	Define any common drive to motoneurons from the RST in healthyhumans	Upper limb muscles (deltoid, biceps brachii, finger flexor, FDI, and SCM.
15	Hammerbeck et al. (2021)	UK	Stroke patients	38	To assess whether the impact of CST and RST connectivity on motor impairment and skill‐acquisition differs in sub‐acute stroke patients, using transcranial magnetic stimulation‐based proxy measures.	Triceps brachii and deltoid.
16	Herbert et al. (2015)	USA	Adult female macaques	2	To create a model of focal cortical ischemia in *Macaca fascicularis* and to explore the contributions of the RST in recovery of reaching.	Pontomedullary reticular formation.
17	Herbert, (2010)	USA	Male monkeys (M fascicularis)	2	Explore the contributions of both the CST and RST in the recovery of reaching following cortical ischemic injury.	ECU, FCR, biceps brachialis, triceps, ADLT, MDLT, PDLT, UTR, MTR, PMJ, and LAT.
18	Honeycutt et al. (2013)	USA	Healthy humans	17	To obtain evidence supporting the hypothesis that the RST has synaptic connections with intrinsic hand muscles in humans.	SCM and right FDI.
19	Li et al. (2014)	USA	Hemiplegic chronic stroke patients	16	To examine reticulospinal excitability at different stages of motor recovery in patients with chronic stroke using the acoustic startling reflex.	Bilateral biceps brachii.
20	Li et al. (2017)	USA	Stroke patients and healthy controls	25 (Stroke: 13; Controls: 12)	To quantify the effect of a startling acoustic stimuli on maximal and sub‐maximal voluntary elbow flexion on the contralesional (impaired) side in stroke survivors as compared to the ipsilesional (non‐impaired) side and healthy controls.	Biceps brachii of both dominant and non‐dominant sides.
21	Maitland & Baker, (2021)	UK	Healthy younger and older adults	83	Assessing reticulospinal and corticospinal contributions to age‐related muscle strength losses in healthy younger and older adults.	Bilateral biceps brachii.
22	Riddle et al. (2009)	UK	Adult rhesus macaque monkeys	3	To examine the synaptic connections between the RST and antidromically identified cervical ventral horn motoneurons, focusing in particular on motoneurons projecting distally to wrist and digit muscles.	Longitudinal fasciculus of the medulla and target muscles: triceps, forearm (wrist and digit) extensors and flexors, intrinsic hand muscles (Interosseus, thenar and hypothenar eminences, and lumbricals 1 and 2).
23	Sangari & Perez, (2019)	Chicago	SCI patients and healthy controls	45 (SCI: 30, controls: 15)	Examining the contributions of the CST and RST for the control of a spastic muscle in humans with chronic incomplete SCI.	Quadriceps femoris muscle.
24	Sangari & Perez, (2020)	USA	Adults with and without chronic cervical incomplete SCI	33 (SCI: 18; controls: 15)	To evaluate whether reduced corticospinal input to triceps brachii and increased reticulospinal input to biceps brachii contribute to the asymmetrical recovery observed in people with cervical incomplete SCI.	Biceps and triceps brachii.
25	Schucht et al. (2002)	Switzerland	Adult rats	45	Relate functional locomotor outcome to the anatomical extent and localization of lesions in the rat spinal cord.	Dorsal, ventral, and ventrolateral funiculus of the spinal cord.
26	Škarabot et al. (2022)	UK	Healthy adults	22	Determine whether the RST modulates maximum force production or maximum motor output.	Vastus lateralis and medialis muscles.
27	Tapia et al. (2022)	UK	Adult female *Macaca mulatta* monkeys	2	To investigate the neural mechanisms underlying the StartReact to provide insights into the interplay between the CST and RST during voluntary movement.	M1, RF, and spinal cord C5‐C8 segments.
28	Tazoe & Perez, (2017)	USA	Healthy volunteers	33	To examine motor evoked potentials elicited by cortical and subcortical stimulation of corticospinal axons (MEPs and CMEPs, respectively) and the activity in intracortical circuits and spinal motoneurons in an intrinsic hand muscle during index finger abduction (control task), precision grip, and power grip.	FDI and the abductor pollicis brevis in the dominant hand.
29	Valls‐Solé et al. (1999)	UK	Healthy participant	14	Identifying the underlying mechanism of startle‐induced reaction time shortening	Wrist extensors, wrist flexors, biceps brachii and triceps brachii muscles, and SCM.
30	Weishaupt et al. (2013)	Canada	Lewis rats, lesioned versus healthy	26 (lesioned: 21; controls: 5)	To investigate whether the spared RST can compensate for the loss of CST input and whether the RST projections rearrange in response to cervical SCI.	Brainstem and spinal cord.
31	Xu et al. (2017)	USA	Patients with first‐time ischemic stroke, hemiparesis and healthy controls	68 (patients: 54; controls: 14)	To develop a new paradigm that could measure these two aspects of hand function (strength recovery and motor control) separately and to investigate the relationship between strength and control over the time course of hand recovery following stroke.	Finger muscles.
32	Zaaimi et al. (2012)	UK	Adult Macaca *mulatta monkeys*, lesioned versus healthy monkeys	9 (lesioned: 3; controls: 6)	To examine the connectivity of the reticulospinal and ipsilateral corticospinal pathways to motoneurons following recovery from corticospinal lesions.	Median and ulnar at the wrist; median and ulnar in the upper arm; deep radial nerve at the elbow.

Abbreviations: ADLT, anterior deltoid; APR, automatic postural response; CST, corticospinal tract; ECU, extensor carpi ulnaris; EDC, extensor digitorum communis; FDI, first dorsal interosseous; FDS, flexor digitorum superficialis; FCR, flexor carpi radialis; LAT, latissimus dorsi; M1, primary motor cortex; MDLT, middle deltoid; MDLT, middle trapezius; MEP, motor evoked potential; PMJ, pectoralis major; PDLT, posterior deltoid muscles; RF, reticular formation; RF, rectus femoris; RST, reticulospinal tract; SCI, spinal cord injury; SCM, sternocleidomastoid muscle; TA, tibialis anterior; UK, United kingdom; USA, United States of America; UTR, Upper trapezius.

**TABLE 2:** Role of reticulospinal tract for motor recovery.

S.no	Author, year of publication	Type of population	Measurement undertaken	Intervention	Motor task(s)	Assessment of RST	Finding/outcome/role of RST
1	Alagona et al. (2001)	Healthy subjects and stroke patients	iMEPs and cMEP	No intervention	Contraction of the FDI, and biceps brachii muscles, (>50% of maximum voluntary contraction for controls) At relaxed state for cases	iMEP	Ipsilateral MEPs with a shorter latency (21.83 vs. 23.56 ms, *p* = 0.025) and smaller amplitudes (12 ± 6% vs. 27 ± 9% of maximum, *p* = 0.001), obtained in relaxed FDI in all patients, except 1. Voluntary contraction slightly shortened the latencies (0.93 ms on average) and markedly increased the amplitudes of iMEPs. In healthy adults, iMEP was found only in >50% MVC and had different characteristics from the iMEP of patients.
2	Baker & Perez, (2017)	Healthy controls and SCI patients	Latency or reaction time	No intervention	Index finger abduction, precision grip, and power grip.	StartReact	Mean EMG activity of SCI patients was larger during VSRT compared with VRT (*p* < 0.01) and VART (*p* < 0.02) trials only during a power grip. In SCI patients, startling stimuli shortened the reaction time for power grip but not for precision grip.
3	Ballermann & Fouad, (2006)	Rat	Projection pattern of the RST in uninjured rats versus rats with thoracic spinal cord hemisection after 7 days (short recovery) or after 42 days (long recovery) Open field locomotion assessed by Beattie and Bresnahan locomotor score Rat's performance monitored by digital video camera	Lateral thoracic hemi section of the spinal cord	Rats walked on a 1‐m‐long horizontal metal bar in weekly testing sessions	Iontophoretic injection of the anterograde tracer biotin dextran amine into the gigantocellular division of the medullary reticular formation	After the long‐recovery period, spared RST fibers sprouted below the injury (at L2). Recovery occurred in parallel with increased numbers of collaterals of spared RST fibers entering the intermediate lamina below the injury at L2. No sprouting of injured RST fibers above the lesion. The degree of the observed changes in sprouted RST collateral density was correlated with improved locomotion (*r* ^2^ = 0.65, *p* < 0.05).
4	Choudhury et al. (2019)	Stroke patients and healthy control subjects	Reaction time (latency)	No intervention	Rapid wrist flexion	StartReact	The RST gain was significantly larger in stroke patients than healthy participants (78.4 vs. 45.0 ms, *p* < 0.05). StartReact effect (VSRT‐VART) was enhanced (170 ms) in some patients with severe damage to CST but not in patients with mild impairment (43 ms).
5	Coppens et al. (2018)	Stroke patients and healthy controls	Reaction time (latency)	No intervention	Voluntary ankle dorsiflexion movements	StartReact	Onset of dorsiflexion was faster during startling stimuli than non‐startling stimuli in both non‐paretic leg of stroke patients (103 ± 17.8 vs. 138 ± 13.7 ms) and in controls (103 ± 17.7 vs. 139 ± 14.6 ms, *p* < 0.001) StartReact accelerated ankle dorsiflexion movements to a similar extent in the paretic (120 ± 45.4 vs. 156 ± 25.3 ms) and non‐paretic leg (*p* < 0.001) Weak APR inter‐ and intra‐limb muscle coordination in stroke subjects substantially improved by StartReact.
6	Darling et al. (2018)	Rhesus monkeys (*Macaca mulatta*)	CRP strength and the number of synaptic boutons in the gigantocellular reticular nucleus of the medulla was estimated	Lesions were made at different regions of the somatosensory and motor cortex		High‐resolution anterograde tracers injected into the arm/hand area of M2 to assess CRP strength, number of synaptic boutons in the Gigantocellular reticular nucleus of the medulla estimated by stereology	Monkeys with lesions of the arm/hand area of M1, lateral premotor cortex, primary somatosensory cortex, and anterior parietal cortex, had twice as many CRP boutons as the controls and monkeys with lesions of the arm/hand area of M1 and lateral premotor cortex (*p* = 0.0002). Total CRP bouton numbers were similar in controls and monkeys with lesions of the arm/hand area of M1 and lateral premotor cortex. Recovery of reaching was strongly correlated with estimated numbers of CRP boutons in monkeys with lesions of the arm/hand area of M1, lateral premotor cortex, primary somatosensory cortex, and anterior parietal cortex.
7	Engmann et al. (2020)	Female lewis rats	Number of sprouting or rewiring/over growth of spared axons	Unilateral hemi section of the spinal cord to destroy or make a lesion in the RST.	Joint movements and limb kinetics during overground locomotion	Adeno‐associated virus injected into specific sites in the brainstem and spinal cord to identify the number of sprouting	Recovery was accompanied by local rewiring and overgrowth of spared axons in the region of the RST.
8	Herbert et al. (2015)	Adult female macaques.	Upper limb motor outputs from right and left cortical motor areas and from the pontomedullary reticular formation were mapped	Focal lesion in the elbow representation of left primary motor cortex created by using Endothelin‐1. Intensive rehabilitative training given for 12 weeks	Reaching activity	Electrodes placed in the PMRF to map upper limb motor outputs from pontomedullary reticular formation by repetitive macrostimulation	Increased in right arm responses from right pontomedullary reticular formation and paucity of left arm responses from left pontomedullary reticular formation (*X* ^2^ = 14.52, *p* = 0.0001). Increased reliance on the pontomedullary reticular formation motor outputs for recovery of voluntary upper limb motor control after significant cortical lesion.
9	Herbert, (2010)	Macaque Monkeys	EMG responses assessed. Reaching was assessed before and after lesion.	Focal ischemic injury induced in the elbow representation of left primary motor cortex by endothelin‐1. Intensive rehabilitation given	Bilateral reaching task	Mapping of upper limb motor outputs from PMRF	After severe lesion, with intensive rehabilitation, gross reaching recovered in a few weeks, and reaching times were slow but comparable to pre‐injury levels by 16 weeks postinjury. Elbow representation in the affected M1 completely absent after recovery, little change in the contralesional motor cortex. Greater right arm representation from left PMRF.
10	Sangari & Perez, (2020)	Adults with and without chronic cervical incomplete SCI	MEP and MVC in biceps and triceps. StartReact and CMEP	No intervention	Isometric elbow flexion and extension	StartReact	Compared with controls, SCI patient had similar MEPs (Controls: MEP‐max = 47 ± 24.3% of the M_MAX_; and SCI: 45 ± 25.7% of the M_MAX_) and MVCs (Controls: biceps = 1.06 ± 0.5 mV, triceps = 0.46 ± 0.2 mV, *p* = 0.03, and SCI: biceps = 0.86 ± 0.4 mV, triceps = 0.26 ± 0.2 mV, *p* = 0.001) in biceps brachii but smaller responses in triceps brachii. In SCI patients, StartReact and CMEP facilitation was larger in biceps brachii but similar to controls in triceps brachii (biceps: 165. ± 38.0% of the CMEP‐test), triceps:118. ±13.6% of the CMEP‐test, *p* = 0.001). CMEP‐startle was larger in SCI compared with controls in biceps (t (22) = 4.7, *p* = 0.001, *d* = 1.6) but not in triceps (t (22) = 0.1, *p* = 0.9, *d* = 0.07). Elbow flexion recovered but not extension.
11	Zaaimi et al. (2012)	*Macaca mulatta* monkeys, lesioned versus healthy monkeys	Intracellular recordings from motoneurons innervating hand and forearm muscles at 6 months post lesion. Recording and comparison of synaptic responses evoked by stimulating the medial longitudinal fasciculus and unlesioned ipsilateral pyramidal tract	Extensive unilateral (left side) lesions of the medullary corticospinal fibers in the pyramidal tract	Finger movement	Intracellular recordings were made from motoneurons innervating hand and forearm muscles when mono‐ and disynaptic excitatory postsynaptic potentials were elicited from the medial longitudinal fasciculus	Input from the ipsilateral pyramidal tract (CST) was rare and weak in both lesioned and control animals. Mono‐ and disynaptic excitatory postsynaptic potentials elicited from the medial longitudinal fasciculus significantly increased in average size after recovery, but only in motoneurons innervating forearm flexor and intrinsic hand muscles, not in forearm extensor muscles.
12	Weishaupt et al. (2013)	Female lewis rats	Quantification of CST and RST	Unilateral ablation of the dorsal CST made at the cervical spinal cord (C4), ventral RST spared, growth stimulating factor administered	Single pellet reaching	Direct count of the RST	Injured animals had made significant improvements in single pellet reaching task at the end of the 6‐week recovery period (20.95% ± 4.96%, *n* = 21; *p* = 0.048), but no sprouting of CST above injury. Injury induced change in density and number in RST above the injury was minor. Overall decrease in RST projections below the injury.

Abbreviations: APR, automatic postural responses; cMEP, contralateral motor evoked potential; CMEP, cervicomedullary motor evoked potential; CRP, corticoreticular projection; CST, corticospinal tract; EMG, electromyography; FDI, first dorsal interosseous; iMEP, ipsilateral motor evoked potential; L2, lumbar vertebra 2; M1, primary motor cortex; Mmax, maximum compound muscle action potential; MVC, maximum voluntary contraction; MEP, motor evoked potential; M2, supplementary motor cortex; PMRF, pontomedullary reticular formation; RST, reticulospinal tract; SCI, spinal cord injury; VART, visual auditory reaction; VSRT, visual startle reaction time.

**TABLE 3:** Role of reticulospinal tract for maximum force production or strength.

S. no	Author, year of publication	Type of population	Measurement undertaken	Intervention	Motor task(s)	Assessment of RST	Finding/outcome/role of RST
1	Anzak, Tan, Pogosyan, & Pogosyan et al., (2011)	Parkinson's disease patient and healthy participants	EMG recording	No intervention	Gripping	StartReact	Improvements in patient's peak rate of force development and the magnitude of force development by loud auditory stimuli (Mean peak yanks (force) increased from 106.5 ± 15.7 kg/s in visual trials to 121.7 ± 17.4 kg/s in startling stimuli, in the OFF‐drug state. Likewise, increases from 105.4 ± 11.5 kg/s to 123.1 ± 13.9 kg/s by startling stimuli ON drug state. In both the cases and controls, there were facilitation of the onset, peak and rate of hand grip force production in response to loud auditory stimulation, over and above that achieved with maximum effort of will.
2	Anzak, Tan, Pogosyan, Djamshidian, et al. (2011)	Healthy participants	EMG recording	No intervention	Gripping	StartReact	Startling stimuli increased peak grip force by 7.2 ± 1.4% (*p* < 0.0001) and Rate of force development by 17.6 ± 2.0% (*p* < 0.00001).
	Colomer‐Poveda et al. (2023)	Resistance‐trained rock climbers and untrained individuals	EMG recording (Rate of force development reaction time)	No intervention	MVC right hand grip contractions	StartReact	Startling stimuli decreased reaction time and increased rate of development and sEMG in both chronic climbers and controls groups (*p* < 0.001). Similar reaction time found during all conditions: visual, non‐startling and SS in both groups. Only SS increased rate of force development compared with the visual (27%, *p* < 0.001, *d* = 0.43 ± 0.50) and non‐startling sound trials (22%, *p* < 0.001, *d* = 0.36 ± 0.50). Greater rate of force development (from 50 to 100 ms) was found in chronic climbers, as compared to the controls, only after startling stimuli (*p* < 0.05, d = 0.85–0.96).
3	Fernandez‐Del‐Olmo et al. (2014)	Trained human subjects	Peak rate of force development, peak velocity, movement onset and duration	No intervention	Concentric bench‐presses	StartReact	As compared to AS (0.017) and VS (*p* = 0.003), the startling SS significantly increased the rate of force development. Significantly higher peak velocity achieved by SS as compared to the AS (*p* = 0.001) and VS (*p* = 0.002). SS resulted in a significant reduction in the movement onset as compared to VS (*p* = 0.0001) and AS (*p* = 0.0001).
4	Glover & Baker, (2020)	Macaque monkey	MEP recorded through chronically implanted EMG electrodes in the upper limb muscles and stimulation of the MLF was used to generate MEP	Resistance training, pulling handle for 3 months; weights added progressively to increase load	Pull a handle with one arm	Stimulating the MLF by implanted electrodes in the MLF	Increased muscle response upon stimulation of the RST. No increase in muscle response during stimulation of CST. Increased synaptic efficacy at reticulo‐interneuron and reticulo‐motoneuron synapses.
5	Glover & Baker, (2022)	Macaque monkeys	Peak firing rate and force recorded from PTNs in primary motor cortex and RF cells.	No intervention	Weightlifting task (0.5‐6 kg weight)	Reticular formation recording done using headpiece incorporated recording chambers	Peak firing rate had significant linear correlation with force for 46.7% of PTNs and 43.5% of RF cells. For almost all RF cells the correlation coefficient was positive Both CST and RST contribute to control of contraction force.
6	Hammerbeck et al. (2021)	Stroke patients	Ipsilesional (CST connectivity) and contralesional (RST connectivity) cortical connectivity assessed. Muscle strength (Motricity Index) measured	Reach training for 3–6 weeks (400 reaches for sufficient task) post‐stroke (plus usual care) versus stroke patients with usual care only	Planar reaching movement	iMEP	At 3 and 12 weeks, strength was associated with ipsilesional (CST) connectivity to the paretic upper limb. AMT for contralateral MEPs was greater when stimulating the affected cortex than the unaffected cortex (56.6 ± 16.7 vs. 42.7 ± 8.0, *p* < 0.001) Strength was not affected by either the presence or absence of contralesional (RST) connectivity.
7	Li et al. (2017)	Stroke patients and healthy controls	EMG and force recordings	No intervention	Ballistic MVC elbow flexion and sub‐maximal voluntary elbow flexion	StartReact	Prevalence of acoustic startle reflex with shorter latency in the impaired biceps brachii was greater than that of the non‐impaired side of stroke subjects and healthy subjects (Impaired side = 62%, non‐impaired side = 28%, and healthy subjects = 14%). During maximal voluntary elbow flexion tasks, the startling stimuli resulted in earlier initiation of elbow flexion (*p* = 0.001) and greater peak torque (*p* = 0.013) in healthy subjects and in stroke subjects with spastic hemiplegia. During sub‐maximal elbow flexion tasks, startling stimuli induced force responses were slightly greater on the impaired side than the non‐impaired side.
8	Maitland & Baker, (2021)	Young and old adults	iMEP and cMEP and their latencies with amplitude were measured. ICAR measured. Association of ICAR with grip strength was assessed	No intervention	Fixed weight resistance task	Using iMEP and ICAR ratio	Significant positive correlation (*r* = 0.43, *p* = 0.0037) between normalized grip strength and ICAR in older adults, but negative significant correlation (*r* = −0.37, *p* = 0.045) in younger adults. Older adults who maintain or strengthen their RST were stronger than their counterparts.
9	Škarabot et al. (2022)	Healthy adults	EMG recording Motoneuron activity was estimated via decomposition of high‐density surface EMG recordings	No intervention	Isometric knee extension (75% of MVC)	StartReact	Reaction time was significantly shorter in response to startling stimuli compared to the visual (*p* < 0.001) and non‐startling sound stimuli (*p* < 0.001). Rate of force development was greater in response to the startling stimuli compared to the other two types of stimuli in the 0–50 ms (33–49%; *p* < 0.001) and 50–100 ms (9–13%; *p* ≤ 0.006) The startling stimuli elicited greater number of discharges per motor unit per second and greater maximal rate of force development.
10	Xu et al. (2017)	Patients with first‐time ischemic stroke and hemiparesis and healthy controls	MVC and individual motor control of each finger assessed five times a year both in stroke patients and controls.	No intervention	MVC of the instructed finger and keeping the uninstructed fingers immobilized	No direct assessment of RST	Recovery of both strength and individual control of fingers appear to occur in the first 3 months following stroke. Corticospinal tract correlated more with individual control of each finger (fine movement control) than with strength.

Abbreviations: AMT, active motor threshold; AS, acoustic stimuli; cMEP, contralateral Motor Evoked potential; CST, corticospinal tract; EMG, electromyography; iMEP, ipsilateral Motor evoked potential; ICAR, ratio of amplitude of Ipsilateral MEP to cMEP; MLF, Medial longitudinal fasciculus; MVC, maximum voluntary contraction; PTNs, pyramidal tract neurons; RF, reticular formation, RST, reticulospinal tract; sEMG, surface electromyography; SAS, startling acoustic stimuli; SS, startling stimuli; VS, visual stimuli.

**TABLE 4:** Role of RST for gross motor function, neural drive and spasticity.

S.no	Author, year of publication	Type of population	Measurement undertaken	Intervention	Motor task(s)	Assessment of RST	Finding/outcome/role of RST
1	Grosse & Brown, (2003)	Healthy subjects	EMG recorded from upper limb muscles	No intervention	Contraction of deltoid, biceps, finger flexors, and FDI, bilaterally upon startle (<50% of MVC)	StartReact	Gross motor function Coherence in the 10–20‐Hz band was significantly greater and above 95% confidence level in the startle reflex than during voluntary tonic contraction for deltoid, but not FDI muscles.
2	Honeycutt et al. (2013)	Healthy humans	EMG recording	No intervention	Abduction of index finger or a grasp task, flexion of fingers at metacarpophalangeal joint.	StartReact	Gross motor function An increased EMG amplitude of grasp task when startle was present while the amplitude of the finger task remained the same (*p* < 0.0001). Startle stimuli resulted in a reduced latency during coordinated grasp but not individual finger movements.
3	Li et al. (2014)	Hemiplegic chronic stroke patients	StartReact responses assessed	No intervention	Rest task, ASR task and 10%, 50%, and 100% of maximum voluntary contraction task	StartReact	Spasticity In subjects without spasticity, StartReact responses were less frequent, 10% on impaired side, and had normal duration of <200 ms. In subjects with spasticity, the responses were more frequent, 58.3% on impaired side, and longer lasting, up to 1 min. Electromyographic activity of the resting nonimpaired limb increased proportionally in subjects with spasticity, but no such correlation in subjects without spasticity.
4	Riddle et al. (2009)	Monkeys	Intracellular recording	Stimulation of descending fibers in the region of the MLF of the medulla	Not reported	Intracellular recording	Gross motor function Significant numbers of motoneurons projecting throughout the upper limb received short latency synaptic input from the RST. Motoneurons received monosynaptic and disynaptic reticulospinal inputs, including monosynaptic excitatory connections to motoneurons that innervate intrinsic hand muscles. Excitatory reticulo‐motoneuronal connections are as common and as strong in hand motoneuron groups as in forearm or upper arm motoneurons. Stimulation of MLF elicited powerful, short‐latency monosynaptic EPSPs (amplitude: 0.81 mV, latency: 0.9 ms) while stimulation of pyramidal tract resulted in a monosynaptic EPSP of shorter amplitude and longer latency (0.6 mV in amplitude, latency: 0.9 ms).
5	Sangari & Perez, (2019)	SCI patients and normal healthy individuals	MEPs, MVCs, and the Start React response	No intervention	Voluntary knee extension (MVC)	StartReact	Spasticity Participants with SCI with spasticity showed smaller corticospinal responses and MVC's and larger reticulospinal gain compared with participants with no or low spasticity and control subjects. Reticulospinal gain was increased in spastic (2.3 ± 0.9) compared with controls (1.8 ± 0.4, *p* < 0.03) and non‐spastic (1.7 ± 0.2, *p* < 0.02) participants.
6	Schucht et al. (2002)	Adult rats	Locomotor outcome was compared with lesion depth, spared total white matter, and spared ventrolateral funiculus	Dorsal and ventral lesions of different severity were made in adult rats	Grid walk	BBB open‐field locomotor score	Gross motor function Preservation of a small number of fibers in the ventral or lateral funiculus was related to stepping abilities and overground locomotion, whereas comparable tissue preservation in the dorsal funiculus resulted in complete paraplegia. The strongest relation to locomotor function was between BBB score and the spared white matter tissue in the region of the RST. Dorsal component containing corticospinal fibers are required for locomotion on the grid.
7	Tapia et al. (2022)	*Macaca mulatta* monkeys	Extracellular recordings from corticospinal neurons in M1, RF, and from the spinal cord C5‐C8 segments	Stimulation of motoneuron pools receiving different proportion of input form the M1 and RF	Elbow flexion/extension movements	Reticular formation recording done using headpiece incorporated recording chambers	Neural drive Startling stimuli suppressed firing rate of cells from M1 (latency: 70–200 ms). However, for the RF cells it increased firing rate (70–80 ms) followed by a significant decrease (140–210 ms). When ≥60% of motoneuron drive derived from RF (≤40% from M1), loud sound shortened reaction time. The extent of shortening increased as more drive came from RF. If RF provided <60% of drive, loud sound lengthened the reaction time.
8	Tazoe & Perez, (2017)	Healthy adults	EMG recording	No intervention	Index finger abduction, precision grip, and power grip	StartReact	Gross motor function A startling stimulus suppressed MEP size during power grip (87.0 ± 20.0%, *p <* 0.05) to a lesser extent than during index finger abduction (62.2 ± 17.8%) and precision grip (78.4 ± 21.8%, *p <* 0.05) and was positively correlated with changes in intracortical inhibition. A startle cue decreased intracortical inhibition, but not CMEPs, during power grip.
9	Valls‐Solé et al. (1999)	Healthy participant	EMG recording	No intervention	Wrist flexion or extension or rising onto tiptoe from a standing position.	StartReact	Neural drive The startling stimulus almost halved the latency of the voluntary response but did not change the configuration of the EMG pattern. In some subjects the reaction times were shorter than the calculated minimum time required for processing of sensory information at the cerebral cortex (the shortening was by more than 70 ms). Most subjects reported that the very rapid responses were produced by something other than their own will.

Abbreviations: ASR, acoustic startle reflex, BBB, Basso, Beattie and Bresnahan; CMEP, cervicomedullary motor evoked potential; CST, corticospinal tract; EMG, electromyography; EPSPs, excitatory post synaptic potential; FDI, first dorsal interosseous; M1, primary motor cortex; MVC, maximum voluntary contraction; MEP, motor evoked potential; MLF, medial longitudinal fasciculus; MVC, maximum voluntary contraction; RF, reticular formation; RST, reticulospinal tract; SCI, spinal cord injury.

### 3.2 Identifying the function of the reticulospinal system

Different experimental animal and human studies examined the function of the RST for motor recovery (12 studies), maximum force production or strength gain (11 studies), and gross motor function and neural drive (seven studies). Further, two studies examined the contribution of the RST for the development of spasticity in stroke and SCI patients.

From a total of 32 human and experimental animal studies, 12 examined the role of the RST for motor recovery. Except for one study (Alagona et al., 2001), all the studies (Baker & Perez, 2017; Ballermann & Fouad, 2006; Choudhury et al., 2019; Coppens et al., 2018; Darling et al., 2018; Engmann et al., 2020; Herbert, 2010; Herbert et al., 2015; Sangari & Perez, 2020; Weishaupt et al., 2013; Zaaimi et al., 2012) revealed that the reticular system had an important role for motor recovery. The majority (seven) of studies examining the function of the RST for motor recovery were nonhuman studies (four on monkeys and three on rat), whereas the other five were among human participants (three stroke and two SCI patients) (Table 2).

Eleven of the 32 studies examined the role of the RST for strength or maximum force production. From the 11 studies, 5 were on healthy participants (Anzak, Tan, Pogosyan, & Brown, 2011; Colomer‐Poveda et al., 2023; Fernandez‐Del‐Olmo et al., 2014; Maitland & Baker, 2021; Škarabot et al., 2022), three on stroke patients (Hammerbeck et al., 2021; Li et al., 2017; Xu et al., 2017), two on nonhumans (i.e., monkeys) (Glover & Baker, 2020; Glover & Baker, 2022) and one on a Parkinson's disease patient (Anzak, Tan, Pogosyan, Djamshidian, et al., 2011). The findings in 10 of the studies (Anzak, Tan, Pogosyan, & Brown, 2011; Anzak, Tan, Pogosyan, Djamshidian, et al., 2011; Colomer‐Poveda et al., 2023; Fernandez‐Del‐Olmo et al., 2014; Glover & Baker, 2020; Glover & Baker, 2022; Li et al., 2017; Maitland & Baker, 2021; Škarabot et al., 2022; Xu et al., 2017) suggested that the RST had a significant role for strength or maximum force production while one study on stroke patients reported that the RST had no role for strength recovery/gain (Hammerbeck et al., 2021) (Table 3).

We identified seven studies (Grosse & Brown, 2003; Honeycutt et al., 2013; Riddle et al., 2009; Schucht et al., 2002; Tapia et al., 2022; Tazoe & Perez, 2017; Valls‐Solé et al., 1999), two on experimental animals, investigating the role of the RST for neural drive and gross motor function. Two studies (Tapia et al., 2022; Valls‐Solé et al., 1999) reported that the reticular system had a role for neural drive for motor tasks while the other five studies demonstrated that the reticular system is important for gross motor function (Grosse & Brown, 2003; Honeycutt et al., 2013; Riddle et al., 2009; Schucht et al., 2002; Tazoe & Perez, 2017) (Table 4). Conversely, two studies (Sangari & Perez, 2019, Li et al., 2014) identified that hyper‐excitability of the RST was a possible cause for spasticity in stroke and SCI patients. The studies were among hemiplegic chronic stroke patients (Li et al., 2014), and incomplete SCI (Sangari & Perez, 2019) patients (Table 4).

## 4 DISCUSSION

Although the RST is known to be responsible for postural control (Mtui et al., 2020), there has been an emergence of new evidence identifying the reticulospinal responses during strength gain and motor recovery (Atkinson et al., 2022; Baker, 2011; Baker et al., 2015). However, there are no reviews that have systematically assessed the role of the RST for strength gain, gross motor function, motor recovery, and spasticity development. Therefore, we aimed to determine the role of the RST by reviewing the body of evidence relating to both human and experimental animal studies. Given the nature of the studies that have examined the RST, it was not feasible to conduct a meta‐analysis (due to heterogeneity), therefore we conducted a scoping review instead. This scoping review identified 32 studies that examined the role of RST and revealed that the excitability of the RST is important for motor recovery, strength gain, and gross motor function. In light of this, there is limited human evidence for the role of the RST for strength gain. Only two studies reported increased RST connectivity in stroke and SCI patients, potentially underpinning spasticity in these populations.

Reticulospinal output is assessed by noninvasive and invasive measures. Invasive measures are used only for experimental animals and it is impossible to use such techniques for humans. The paucity of human evidence concerning the RST primarily stems from the challenge of directly assessing its function through noninvasive stimulation methods. As the reticular system is located deep within the brainstem, it is not feasible to stimulate it directly using techniques such as transcranial magnetic stimulation (TMS) or any other available means (Glover & Baker, 2020). Therefore, other indirect measure become mandatory to elucidate the function of the RST. Two methodologies, the “StarReact” Paradigm and iMEP, have been shown to probe the excitability of the RST in humans. The StartReact paradigm is a simple measure of latency or reaction time to a preplanned action. It involves the simultaneous presentation of two stimuli: an imperative visual stimulus and an unexpected loud sound or startling stimuli (Carlsen & Maslovat, 2019). The visual stimulus serves as a cue for executing a preplanned action or response. The unexpected startle stimulus, transmitted via the cochlear nerve, directly activates the motor nuclei in the caudal pontine reticular formation. Additionally, it indirectly stimulates the reticular formation through the lateral lemniscus, leading to the rapid initiation of the preplanned action (Yeomans & Frankland, 1995). This will add to the corticospinal input, thereby increasing the overall excitatory input to the lower motoneurons which speed the initiation of the preplanned response (Valls‐Sole, 2012; Yeomans & Frankland, 1995). The other noninvasive technique to assess the excitability of the RST is to record iMEPs. The iMEP is elicited by applying single‐pulse TMS over the primary motor cortex with a near maximum or maximum stimulatory output and strong back background muscle contraction (Tazoe & Perez, 2014; Wassermann et al., 1991; Wassermann et al., 1994). Transcranial magnetic stimulation triggers the primary motor cortex, leading to the activation of the cortico‐reticular pathway. These pathways, in turn, stimulate the RST that project bilaterally to the spinal cord, subsequently exciting the lower motoneurons to elicit iMEPs from the corresponding ipsilateral muscle (Fisher et al., 2012; Ziemann et al., 1999). Nevertheless, the elicitation of iMEPs has been observed to be more readily achieved in older adults (Maitland & Baker, 2021), and stroke patients (Alagona et al., 2001). It has proven to be less successful in evoking iMEPs in young and healthy participants (Alagona et al., 2001; Maitland & Baker, 2021). Therefore, the StartReact paradigm appears to be a more effective technique for assessing the excitability of the RST in humans.

### 4.1 Function of the RST for motor recovery

This scoping review demonstrated that the RST underpins motor recovery. Eleven out of 12 studies (Baker & Perez, 2017; Ballermann & Fouad, 2006; Choudhury et al., 2019; Coppens et al., 2018; Darling et al., 2018; Engmann et al., 2020; Herbert, 2010; Herbert et al., 2015; Sangari & Perez, 2020; Weishaupt et al., 2013; Zaaimi et al., 2012) demonstrated increased RST activity suggesting the excitability of the RST is important for motor recovery. For example, a study on SCI patients provided evidence of increased excitability of the RST by the enhanced StartReact CMEP facilitation (StartReact + CMEP) and maximal voluntary contractions (MVCs) in biceps brachii (as compared to the controls), but not in triceps, of SCI patients with recovered elbow flexion but not extension (Sangari & Perez, 2020). These data suggest that during motor recovery of the biceps brachii, there is increased RST input to the biceps brachii to restore and preserve motor function. At a minimum, this suggests that the RST compensates for the spinal cord lesion and provides a neural pathway to innervate the biceps brachii. Interestingly, the absence of recovery in elbow extension could potentially be attributed to the decline in CST drive and diminished input from the RST to the triceps muscle. This discrepancy in recovery may arise from the distinct pattern of innervation by the RST to the elbow flexors and extensors. This line of enquiry is consistent with the shorter reaction time following the startling cue during the power‐grip action of SCI patients (Baker & Perez, 2017). Overall, these findings suggest that increased activity of the RST is important for motor recovery in humans following SCI. The absence of a decrease in StartReact following the precision grip implies that enhanced activity of the RST plays a significant role in gross motor function and recovery, rather than fine motor control. This is most likely as a result of the RST supplying a large group of muscles in a synergistic manner (Peterson et al., 1975) that enables gross function, whereas the CST supplies a smaller group of motoneuron pools (Buys et al., 1986) suited for fine‐grade movement (Zaaimi et al., 2018). In addition to the recovery of the power grip, recovery of muscle coordination has also been reported as the other possible role of RST. For example, in chronic stroke patients, increased RST activity improved automatic postural responses by startling acoustic stimuli. Moreover, startling acoustic stimuli was shown to reduce the latency of automatic postural responses in both stroke patients and healthy controls (Coppens et al., 2018). As the automatic postural responses is a measure of muscle coordination, these preliminary data suggested that the recovery of muscle coordination could be due to the intrinsic arrangement, extensive collaterals, of the RST (Peterson et al., 1975) supplying many motor units and thereby controlling muscle coordination. The above findings in humans are supported by experimental animal studies (Ballermann & Fouad, 2006; Engmann et al., 2020; Zaaimi et al., 2012).

In experimental animals that have recovered following CST lesions, there is evidence to show enhancement and increased size of MLF derived mono and disynaptic excitatory postsynaptic potentials onto motoneurons of intrinsic hand muscles and forearm flexors. Importantly, this was not accompanied by changes in the lesioned ipsilateral pyramidal tract which again implies a limited role of the CST tract for motor recovery, but an important role of the RST for motor recovery of the forearm flexors and intrinsic hand muscles (Zaaimi et al., 2012). Moreover, studies on experimental animals (i.e., female Lewis rats) support this finding. For example, the sprouting of the spared RST below the level of a hemisection at L2 of the spinal cord following 42 days of recovery was observed. In addition, a positive correlation between the density of sprouted spared RST and the degree of locomotor recovery was reported (Ballermann & Fouad, 2006). Other correlations exist between the degree of recovery and pronounced rewiring/plasticity of the injured neurons and the compensatory overgrowth of spared neurons in the gigantocellularis reticularis (Engmann et al., 2020). These data imply an emerging role for the RST to modulate aspects of motor recovery. Contrary to the aforementioned findings, motor recovery in lesioned (unilateral) Lewis rat was found to be associated with a decrease in the number of reticulospinal fibers below the level of the lesion (C4) (Weishaupt et al., 2013). Experimental errors may well account for the observed contradictory findings. Moreover, the difference in site of the induced lesion may have a different outcome of recovery, as the RST has diverse neurotransmitters and projections (Peterson et al., 1975). Therefore, tracing different reticulospinal fibers may perhaps result in a different outcome between experimental animals. Further, it is plausible to propose that inflammatory processes in the vicinity of surviving fibers might adversely influence their growth, resulting in a decrease in reticulospinal fiber density specifically on the side of the lesion. Although the later contradictory study has acknowledged and addressed these conceivable causes of variation, they still have the potential to influence the final outcome.

Two studies, one human study on chronic stroke patients (Engmann et al., 2020), and the other on experimental animal (Herbert et al., 2015), revealed that the recovery role of the RST is affected by the degree of severity of the cortical lesion. Reaction time was faster, showing greater involvement of the reticular system in severely impaired patients than in the mildly affected patients (Choudhury et al., 2019). In the case of the experimental animal (i.e., monkey), with severe lesion of the M1, recovery of a reaching task was achieved after 12‐weeks of intensive rehabilitation training without any ipsi‐lesional and/or contra‐lesional cortical plasticity, excluding the possibility for the CST to be a site for recovery. However, Evidence of spontaneous recovery and cortical plasticity was observed in monkeys with mild lesions in the M1 after a 2‐week period, without any intervention. Conservatively, these data implied that recovery occurs at the level of the cortex. On the contrary, the RST likely modulates motor recovery when the cortical lesion is severe, whereas the surviving CST continues to function and maintain motor function during mild lesions. Therefore, the RST or the reticular system strengthened to compensate for the loss of function of the CST, by maintaining motor function.

In support of the above, there is evidence to suggest that the RST increases its excitability, a mechanism associated with plasticity. For example, experimental animals (i.e., Rhesus monkeys) with lesions to the M1, lateral premotor cortex, primary somatosensory cortex (S1), and anterior partial cortex exhibited elevated activation levels of the cortico‐reticular projection. This projection originates from the supplementary motor cortex and targets the reticular formation in the medulla. Furthermore, the lesioned monkeys showed an increase in the total number of cortico‐reticular projection buttons within the reticular formation gigantocellularis when compared to monkeys without lesions, serving as control subjects. Furthermore, the number of cortico‐reticular projection buttons was strongly correlated with the degree of motor recovery of the hand (Darling et al., 2018). Likewise, intensive rehabilitative training in lesioned monkeys resulted in recovery of gross reaching by the 16th week with a representation of right arm at the left pontomedullary reticular formation, while no recovery of arm representation was observed at the lesioned M1 (Herbert, 2010). Similarly, reaching was recovered substantially without recovery of the contra or ipsi‐lesional cortical representation in monkeys (*Macaca fascicularis*) with severe lesion to their M1 after 12‐weeks of intensive rehabilitation training (Herbert et al., 2015). These findings provide evidence for the possibilities for the reticular system/RST to be the site for motor recovery in clinical populations including stroke and SCI patients. Therefore, targeting the connectivity of the RST with specific neurorehabilitation training could be the key to treatment for improving motor recovery.

Overall, in regard to determining the site and mechanism underlying motor recovery following injury, many of the studies imply that neuroanatomical plasticity of the reticular system occurs specifically at the gigantocellularis reticularis. However, a single study on lesioned female Lewis rat (Weishaupt et al., 2013) revealed that motor recovery is not the result of anatomical change, but rather a change in plasticity at cellular level. The authors reported that the improvement in a single pellet reaching task by Week 6 was accompanied by only a minimal increase in density and number of RST projections without sprouting of CST projection beyond the level of injury and decreased RST projection below the level of the injury. This suggests that other mechanisms could be involved, such as increased firing rates of neural cells at the reticular formation and RST activation.

In contrast, only one study (Alagona et al., 2001) reported that the RST had no role for motor recovery in acute stroke patients. It stated that the source of the iMEP, which was a good prognostic indicator of motor recovery in acute stroke patients by the sixth month, was the hyperactivated premotor area, whereas the cortico‐RST was the source of iMEP for healthy participants. However, we suggest that the source of iMEP for the stroke patients might be the activated cortico‐reticular projection from the hyperactivated supplementary motor cortex (Li et al., 2019). Furthermore, because of the bilateral nature of the RST, it was suggested that increased connectivity of the RST, occurs post stroke to preserve motor function and to act as an accessory motor pathway to compensate for the loss of function of the CST (Li et al., 2019).

### 4.2 Role of RST for strength gain

Based on the limited experimental animal and human studies, the reticular system seems to have an important role for strength gain. The findings in two experimental animals and eight human studies (Anzak, Tan, Pogosyan, & Brown, 2011; Anzak, Tan, Pogosyan, Djamshidian, et al., 2011; Colomer‐Poveda et al., 2023; Fernandez‐Del‐Olmo et al., 2014; Glover & Baker, 2020; Glover & Baker, 2022; Li et al., 2017; Maitland & Baker, 2021; Škarabot et al., 2022; Xu et al., 2017) suggest that RST activity is important for the expression of strength or maximum force production. For example, four studies on healthy adults (Anzak, Tan, Pogosyan, & Brown, 2011; Colomer‐Poveda et al., 2023; Fernandez‐Del‐Olmo et al., 2014; Škarabot et al., 2022) revealed that startle stimuli, which activates the RST, resulted in a shorter reaction time, increased motor discharge per motor unit per second (maximum motor output), increased rate of force development and greater force production, when compared to the visual acoustic and visual only stimuli. Moreover, a cross‐sectional study on healthy humans (Maitland & Baker, 2021) showed that older adults with better strength had greater RST connectivity than weaker, older adults. In support of these findings in healthy participants, two studies on stroke patients (Li et al., 2017; Xu et al., 2017) and one study on a Parkinson's disease patient (Anzak, Tan, Pogosyan, Djamshidian, et al., 2011) reported a role of the RST for strength gain. For example, startling acoustic stimulus resulted in a shorter reaction time and induced greater force generation in the impaired biceps brachii when compared to the non‐impaired biceps brachii of stroke patients and healthy controls (Li et al., 2017). The startling stimulus elicited a greater increase in force production on the impaired side compared to the non‐impaired side. Furthermore, in ischemic stroke patients with lesion to the hand area of the M1, the excitability of the CST was not correlated with force production, but rather, was correlated with individual motor control of each finger (individuation). This finding suggests the presence of other tracts, possibly the RST, responsible for the restoration of muscle strength and contributing to force production (Xu et al., 2017). In support of this, a loud startling stimulus was found to increase peak rate and magnitude of force development in a Parkinson's disease patient (Anzak, Tan, Pogosyan, Djamshidian, et al., 2011).

The findings in human studies are supported by monkey studies; for example, strength gain after resistance training in two Macaque monkeys was accompanied by a post‐training increase in RST excitability, but there was a variable change in the excitability of the CST (Glover & Baker, 2020). This increased RST output was attributed to increased synaptic efficacy at the level of the (monosynaptic) reticulo‐motoneuron and (di‐synaptic connection) reticulo‐inter neuron levels. Overall, the RST's bilateral nature (Davidson et al., 2007), its extensive collaterals, and high degree of divergence, appear to enable the coactivation of several muscles in synergistic patterns (Peterson et al., 1975), making the RST well suited to modulate maximum force production and execution of forceful movements, thereby modulating strength gain.

Both the RST and CST were found to contribute to force generation, with the RST being important for force production and the CST being important for fine‐force scale adjustment (Glover & Baker, 2022). These distinct functions are likely to be attributed to the neuroanatomical nature of these tracts, that is, the RST supplies large group of muscles (Peterson et al., 1975) well‐matched for force production while the CST controls small group muscles or small motor units (Buys et al., 1986) appropriate for controlling fine movements. Finally, one study among stroke patients reported that the presence or absence of RST connectivity had no effect on strength, rather CST connectivity was found to be responsible for force production (Hammerbeck et al., 2021). This difference could be attributed to the difference in the target muscle or nerve used to assess the contribution of the RST and CST for strength or maximum force production. Triceps and deltoid muscle were used as target muscles by the latter study, which reported that the RST had no role for strength. The RST has been shown to have less contribution to elbow extensor connectivity (Sangari & Perez, 2020).

### 4.3 Role of RST for gross motor function and neural drive

It has been also reported that the reticular system has a role for neural drive for motor activities (Tapia et al., 2022; Valls‐Solé et al., 1999) and gross motor function (Grosse & Brown, 2003; Honeycutt et al., 2013; Riddle et al., 2009; Schucht et al., 2002; Tazoe & Perez, 2017). Experimental evidence indicates that the reduction in latency observed during StartReact, which is even shorter than the time required for sensory processing, strongly suggests extensive activation of the reticular formation and an amplification of motoneuron activation (Valls‐Solé et al., 1999). Further, this line of inquiry is supported by experimental animal studies. The reaction time in monkeys was shown to be shortened by the startling stimuli when neural drive was derived (>60%) from the reticular formation but lengthened when the neural drive was derived (>60%) from M1 (Tapia et al., 2022). These findings highlight the possibility for the reticular system to be, at least in part, the site for storage of instructions for movement. During StartReact, the motor instruction to achieve the movement goal will possibly be stored in the brain stem and will be triggered rapidly and automatically in response to the startle or loud sound (Rothwell, 2006).

Synchronization of the bilateral homologous muscles of the proximal upper limb muscles was the other reported function of the RST which was evidenced by the synchronization of EMG recordings that were significantly higher or had better coherence during startle than voluntary contraction alone, between homologous bilateral muscles in biceps brachii but not in first dorsal interosseous muscle (Grosse & Brown, 2003). The involvement of the reticular system for the synchronization of the bilateral homologous muscle is because of the neuroanatomical features of the RST. It has highly divergent postsynaptic connections (Riddle et al., 2009) thereby innervating many motor units allowing it to control coordination and synchronization of homologous muscles.

The findings above have been confirmed in nonhumans rats. Lesions made at different sites of the brainstem and sparing of small white matter tissue in lateral and medial funiculus, the site where the RST originates, preserves movement, whereas sparing of a comparable mass of tissue at the dorsal funiculus resulted in paraplegia (Schucht et al., 2002). In addition, MEPs, when obtained during the StartReact paradigm, are suppressed to a lesser extent during a power‐grip task than during a precision‐grip task (Tazoe & Perez, 2017). In experimental animals (monkey), the extensive number of excitatory synaptic inputs from the reticular system to motoneurons of a hand muscle upon stimulation of the MLF in monkeys (Riddle et al., 2009) provides evidence for the involvement of the RST in gross motor control of the hand. Similar observations have been made in humans, consistent with the findings in monkeys. It has been reported that the RST contributes input to intrinsic hand muscles, facilitating the control of coordinated gross hand movements or whole hand movements, rather than individual finger movements (Honeycutt et al., 2013). Taken together, these findings in experimental animals and human studies provide emerging evidence which confirms the role of the RST for gross hand function. The role of the RST in gross function is because it connects large groups of muscles in a synergetic manner that enables gross function (Peterson et al., 1975), whereas the CST supplies small groups of motoneuron pools (Buys et al., 1986) suited for fine‐grade movement (Zaaimi et al., 2018).

Even though most of the studies revealed that the RST has an important role for motor recovery, strength, and gross functions or movements, hyper‐excitability of the RST was purported to be the possible cause of spasticity in stroke and SCI patients (Li et al., 2014; Sangari & Perez, 2019). The presence of an exaggerated and prolonged acoustic startle reflex (lasting up to 1 min) in the spastic biceps brachii, coupled with a reduced frequency compared to the normal acoustic startle reflex in the non‐spastic impaired limb of hemiplegic chronic stroke patients, provides compelling evidence for the potential involvement of RST hyper‐excitability as the underlying cause of spasticity (Li et al., 2014). Increased EMG activity of the unaffected limb of a patient with spasticity (compared to non‐spasticity patients) was thought to be attributed to contralateral overflow of the hyperexcitable RST (Sangari & Perez, 2019). Similarly, SCI patients with spasticity (compared to controls and non‐spasticity patients) were found to have shorter reaction time which correlated with the degree of spasticity, less corticospinal response, larger StartReact, larger maximum voluntary contraction and reticulospinal gain. This finding implies that the spasticity in stroke and SCI patients can be attributed to the hyper‐excitability of the reticulospinal system leading to increased excitability of muscle stretch reflexes or muscle tone.

### 4.4 Limitations

Included studies were not assessed for quality which could potentially impact the robustness of our conclusions, but it is important to note that quality assessment is not a requirement for scoping reviews. The variations in RST function, assessment technique and type of population, the presence or absence of an intervention, and variation in duration of intervention among studies are further limitations that should be considered. Lastly, the inclusion of experimental animal studies restricts the generalizability of findings to humans; however, their incorporation was crucial in illustrating the physiological aspects of the RST underpinning motor recovery, strength gain and other roles of the RST.

## 5 CONCLUSIONS AND FUTURE DIRECTIONS

The overall findings of this scoping review suggest that the RST has an important role in motor recovery. Therefore, the RST is a promising target for neurorehabilitation enabling stroke and SCI patients to recover successfully. Based on a limited number of studies, it appears that the connectivity of the RST, at least in part, underpins strength gain and force production. Moreover, the excitability of the RST is important for the control of gross motor function, neural drive, and the spasticity in stroke and SCI patients might be attributed to the hyper‐excitability of the RST.

Further research is necessary to obtain more robust evidence and gain a comprehensive understanding of the role of the RST in strength enhancement and force production in humans. To better comprehend the sites and neural mechanisms underlying strength gains and motor recovery, future studies should focus on investigating the entire neural axis or specifically the cortico‐RST. In particular training interventions that target the RST, such as coupling a loud acoustic stimulus with TMS or adding acoustic stimuli during strength training, may enhance the excitability of the RST. In addition, the way in which the strength training is performed, for example paced versus self‐paced may lead to different neural adaptations, with paced strength training leading to a corticospinal tract response, while self‐paced training may increase the excitability of the RST. However, there are no studies to date that have examined this hypothesis. Further, additional studies are needed to explore the involvement of the RST in the development of spasticity in individuals with SCI, and stroke patients, and other conditions resulting in spasticity. These investigations have the potential to yield compelling evidence and hold significant implications for designing targeted strength training and neurorehabilitation programs. Such interventions can promote strength improvements in the general population, facilitate successful motor function recovery in patients, and enhance performance in sport‐specific activities.

## AUTHOR CONTRIBUTIONS

Yonas Akalu, Ashlyn K Frazer, Jamie Tallent, Alan J. Pearce and Dawson J Kidgell: Contributed to Conceptualization, Methodology, Data curation, formal analysis, writing‐original preparation. Glyn Howatson, Ummatul Siddique and Mohamad Rostami: Writing‐review and editing. All authors approved the final manuscript.

## FUNDING INFORMATION

This project was funded by the Advancing Women's Research Success Grant (Monash University 2020–2021) awarded to Dr Ashlyn Frazer.

## CONFLICT OF INTEREST STATEMENT

The authors declared no conflict of interest.

## Supporting information


Data S1.
