# Supplementary material for: Identifying the role of the reticulospinal tract for strength and motor recovery: A scoping review of nonhuman and human studies
Source: Physiol Rep. 2023 Jul 20;11(14):e15765. doi: 10.14814/phy2.15765 (PMC10359156; doi:10.14814/phy2.15765)
Supplement: Supplementary file 1 — Data S1. [file PHY2-11-e15765-s001.docx]

**Supplementary File 1: Search strategies utilised**

1. **Search strategy for OVID Medline database**

(("Reticulospinal tract*" OR "Reticulospinal outflow*****" OR" Ipsilateral motor-evoked potential*" OR "Ipsilateral MEP*" OR "Acoustic startle*" OR "StartReact"*****) and (("Hand strength*" OR "Muscle strength*" OR "Muscle force*" OR "Maximum voluntary contraction*" OR "Balance*" OR "Motor Recover*" OR "voluntary elbow flexion*" OR "Grip strength*" OR "Sarcopenia" OR "Isometric contraction*" OR "wrist flexor muscle*" OR "hand muscle*" OR "dorsal interosseous muscle*")

1. **Search strategy for Embase database**

(("Reticulospinal tract*" OR "Reticulospinal outflow*****" OR" Ipsilateral motor-evoked potential*" OR "Ipsilateral MEP*" OR "Acoustic startle*" OR "StartReact"*****) and (("Hand strength*" OR "Muscle strength*" OR "Muscle force*" OR "Maximum voluntary contraction*" OR "Balance*" OR "Motor Recover*" OR "voluntary elbow flexion*" OR "Grip strength*" OR "Sarcopenia" OR "Isometric contraction*" OR "wrist flexor muscle*" OR "hand muscle*" OR "dorsal interosseous muscle*")

1. **Search strategy for CINAHL database**

("Reticulospinal tract*" OR "Reticulospinal outflow*" OR "Reticulospinal contribution*" OR" Ipsilateral motor?evoked potential*" OR "Ipsilateral MEP*" OR "Acoustic startle*" OR "StartReact"*) AND ("Hand strength*" OR "Muscle strength*" OR "Muscle force*" OR "Maximum voluntary contraction*" OR "Balance*" OR "Motor Recover*" OR "voluntary elbow flexion*" OR "Grip strength*" OR "Sarcopenia" OR "Isometric contraction*" OR "wrist flexor muscle*" OR "hand muscle*" OR "dorsal interosseous muscle*")

1. **Search strategy for SCOPUS database**

**( ( TITLE-ABS-KEY ( reticulospinal  AND  tract )  OR  TITLE-ABS-KEY AUTH ( ipsilateral  AND  motor  AND  evoked  AND  potential )  OR  TITLE-ABS-KEY ( acoustic  AND  startle  AND  reflex )  AND  TITLE-ABS-KEY ( acoustic  AND  startle  AND  response ) )  AND  ( TITLE-ABS-KEY ( hand  AND  strength )  OR  TITLE-ABS-KEY ( muscle  AND  strength )  OR  TITLE-ABS-KEY ( muscle  AND  force )  OR  TITLE-ABS-KEY ( maximum  AND  contraction* )  OR  TITLE-ABS-KEY ( balance )  OR  TITLE-ABS-KEY ( motor  AND  recover* )  OR  TITLE-ABS-KEY ( voluntary  AND  elbow  AND  flexion )  OR  TITLE-ABS-KEY ( isometric  AND  contraction )  OR  TITLE-ABS-KEY ( grip  AND  strength )  OR  TITLE-ABS-KEY ( sarcopenia )  OR  TITLE-ABS-KEY ( adaptation ) ) )**
